# Exome sequencing in genetic disease: recent advances and considerations

**DOI:** 10.12688/f1000research.19444.1

**Published:** 2020-05-06

**Authors:** Jay P. Ross, Patrick A. Dion, Guy A. Rouleau

**Affiliations:** 1 Department of Human Genetics, McGill University, 3640 University, Montréal, QC, H3A 0C7, Canada; 2 Montreal Neurological Institute and Hospital, McGill University, 3801 University, Montréal, QC, H3A 2B4, Canada; 3 Department of Neurology and Neurosurgery, McGill University, 3801 University, Montréal, QC, H3A 2B4, Canada

**Keywords:** whole exome sequencing, disease research, whole genome sequencing

## Abstract

Over the past decade, exome sequencing (ES) has allowed significant advancements to the field of disease research. By targeting the protein-coding regions of the genome, ES combines the depth of knowledge on protein-altering variants with high-throughput data generation and ease of analysis. New discoveries continue to be made using ES, and medical science has benefitted both theoretically and clinically from its continued use. In this review, we describe recent advances and successes of ES in disease research. Through selected examples of recent publications, we explore how ES continues to be a valuable tool to find variants that might explain disease etiology or provide insight into the biology underlying the disease. We then discuss shortcomings of ES in terms of variant discoveries made by other sequencing technologies that would be missed because of the scope and techniques of ES. We conclude with a brief outlook on the future of ES, suggesting that although newer and more thorough sequencing methods will soon supplant ES, its results will continue to be useful for disease research.

## Introduction

The genetic view of disease etiology has historically focused on finding a causal variant for a given phenotype. This approach has worked well for diseases that are ostensibly monogenic, such as cystic fibrosis ^1^ or Huntington’s disease ^2^. Within a pedigree, the segregation of genetic variants with a given phenotype was originally studied using linkage analysis ^3^. Though instrumental for finding associations of simple genetic factors with disease, early linkage studies typically needed further experiments in fine mapping disease loci in order to find a candidate protein-altering variant within a gene.

Inherited or acquired protein-coding variants represent the majority of disease-causing variants, accounting for upwards of 60% of all known causative genomic variation ^4, 5^. Exome sequencing (ES) is the targeted sequencing of nearly every protein-coding region of the genome ^6, 7^. Typically, either a hybridization capture or multiplex primer-based amplification is used to generate libraries of exonic sequences that can be mapped to the reference genome to find variants. Given the abundance of knowledge on protein-coding genes compared with other regions of the genome, ES leverages well-sequenced and mapped regions of the genome with *in silico* predictions of protein function. The field of genetics was shifted from multi-step loci discovery and subsequent resequencing to testing nearly every protein-coding gene simultaneously.

The number of diseases and syndromes that are explained by a single variant, or even a single altered gene, becomes smaller and smaller as the field of genetic research progresses. Indeed, even diseases easily defined as monogenic are now being studied in terms of genetic modifiers of severity and age at onset ^8– 10^. Monogenic or “familial” forms of a heterogenous disease often account for small proportions of a total disease population ^3^. Taking as an example the genetic etiology of amyotrophic lateral sclerosis (ALS), a multi-step model has been proposed to incorporate risk from genetic variants and environmental exposure ^11^. In this model, an apparently monogenic variant would account for several or all “steps” that are necessary to instigate disease. However, the same disease might be acquired through several variants of lower penetrance or through a combination of genetics and environmental factors.

In this review, we will outline the recent successes and applications of ES and subsequent gene discovery in disease research. We aim to demonstrate the utility and efficacy of ES while giving a perspective on the future of the study of genetic disease etiology, specifically focusing on upcoming techniques and technologies. While ES has been instrumental in broadening our knowledge of disease genetics, the lessons learned from ES studies will bridge the gap into genome sequencing (GS), long-read sequencing (LRS), and beyond.

## Exome techniques and discovery examples

### Early disease gene discovery using exome sequencing

Early uses of ES in gene discovery focused mainly on segregation of a variant or variants within a gene and the phenotype of interest. Generally, a family containing multiple affected individuals would be subjected to ES in order to find variants that are observed only in affected cases and not in the unaffected relatives or spouses ^12^. Candidate variants that segregated well with the disease phenotype would be screened in other families with the intent to reproduce the same degree of segregation. ES could combine the unbiased approach of genome-wide linkage with the direct observation of protein-altering variants as in Sanger sequencing exons of a candidate gene ^12^. Examples of successful discoveries using this paradigm include rare inherited forms of ALS ^13– 15^, Parkinson’s disease ^16^, epilepsy ^17^, and heart diseases ^18, 19^. These examples illustrate the efficiency of ES applied to diseases that are caused by or associated with penetrant and monogenic variants, but it is considerably more analysis-intensive for variants with variable penetrance or private variants observed in only a single pedigree ^20^.

## Clinical exome sequencing

Genetic studies are dependent on the accuracy of diagnosis, the pleiotropy of variants of a given gene, and the prior association of variants with an outcome. Determining a specific diagnosis can be challenging because several diseases and disorders can have similar or overlapping symptoms ^21^. Pleiotropic genes, whose variants can result in a variety of diseases, can also lead to lower diagnostic efficacy ^22^. Therefore, genetically heterogenous diseases with many known genetic causes benefit more from an unbiased screening of all genes, as in ES or GS ^23^.

ES is now used as a rapid and effective means to diagnose or aid in the diagnosis of disease. ES can be employed at a prenatal period to detect fetal abnormalities ^24– 26^ and postnatally following a phenotypic observation ^27, 28^. A significant benefit of ES comes from the ability to determine whether genetic abnormalities have been inherited from the parents or whether a variant has occurred spontaneously during gametogenesis or in gestation ( *de novo* or genetic mosaicism, respectively) ^29^. This screening could help to inform current medical intervention or to act as a basis for genetic counselling. While the utility of genetic aid in diagnosis is not limited to ES (GS and panel sequencing are also used), the cost, speed, and ease of interpretation of ES maintain it as a preferred method ^30^. As a result of the combined knowledge generated from association studies and further functional validation of variants, the ability to generate diagnoses from ES data will increase. For example, given an uncertain phenotype in a newborn, success rates to provide a diagnosis through ES continue to rise, between about 50 and 80% of all cases depending on phenotypic severity and the range of diagnoses ^24, 28^. The success rate of clinical ES appears to lower for older patients with adult-onset phenotypes, between about 25 and 50% ^31, 32^; the difference in success rate because of age could be due to several factors ranging from probands being presymptomatic until later in life ^32^ to the amount of research performed on different diseases.

The application of ES is dependent on the direction in which the technology is used. Applying ES on a patient with non-specific symptoms or a novel disorder might not be as informative as for a patient with a well-characterized disease with many uniquely associated genes. The average ES results of a patient can generate more than 20,000 variants, of which a median of 21 would be predicted as loss of function ^33^. Determining the genetic cause of a rare disease proves difficult as observed variants in an individual may be either coincidental or, if truly causal, private to a family. Interpretation of these variants is critical to filtering out common variants and prioritizing candidate variants.

Whether testing during the pre- or post-natal period or testing relatives of a proband for potentially associated variants, there is always the risk of incidental genetics findings. Variants are sought after clinically for an involvement in the phenotype or disease of interest, but as ES allows the discovery of potentially all coding variants, this can result in the discovery of variants that are not related to the clinical testing ^34^. Additionally, incidental issues with relatedness testing can arise ^32^ and could affect both clinical efficacy and personal aspects. As ES data become accessible from open-science initiatives and data sharing across consortia, reanalysis of data should also be undertaken following qualified guidelines (for example, from the Canadian College of Medical Geneticists) ^35^. New techniques and analysis paradigms will continue to be generated, and ES data should be reanalyzed with the newest information in order to best utilize the data generated ^36^. As ES is also expanded into industry settings, ethical care and legal preventions must be taken to avoid the improper dissemination of incidental findings to patients and customers alike.

## *De novo* variants and pooled-parent exome sequencing

Genetic variants that are not present in the genomes of the parents of the proband arise *de novo*. ES of both unaffected parents and the affected individual, known as “trio sequencing”, has been successful to find genes in which these *de novo* variants are associated with disease ^21, 37– 39^. Although certain specific disease-related variants tend to arise *de novo*, such as the p.P525L variant in *FUS* associated with ALS ^40, 41^, an unbiased approach to finding *de novo* variants is required in most cases.

A recent innovation in the detection of *de novo* variants has been the “pooled parent” approach ^42^. Because detection of *de novo* variants necessitates knowing the genetic status of both parents of a proband, sequencing can be more costly than simply screening a proband with ES. By fully sequencing the proband with ES, detailed and high-fidelity information about variants is acquired; as parents are used only to filter out non- *de novo* variants, less information is required on their genetic status. Therefore, in this method, the parents of all sequenced probands are pooled into a single ES sample and used to test for the presence of any candidate *de novo* alleles in the parent generation. The impetus for this approach was to use the lower cost of the singleton approach (that is, screening only the proband for likely causal variants) while using a population of parent genomes to increase diagnostic yield. However, the necessity of collecting and sequencing parental DNA applies to both traditional trio sequencing and pooled-parent sequencing, which is often a difficult task in late-onset disease research. The study also uses the gnomAD public database ^43^ to filter variants that are observed in a significant proportion of the general population and likely not a *de novo* cause of disease ^42^. However, as not every ethnicity or population is represented in gnomAD, it is essential to sequence both parents of a given proband in the pooled-parent technique, as the absence of a variant in gnomAD is not adequate to conclude that a *de novo* variant has occurred.

The pooled-parent approach to ES demonstrates that an established technique can be refined. Although the same conclusions are reached after applying traditional or pooled-parent trio sequencing, a larger diagnostic yield can be achieved, enabling lower-cost and more efficient processing of genetic analysis.

## Studying rare variation

Many exome variants have a low allelic frequency in the general population. If a variant alters the coding sequence of a protein (that is, if the variant is non-synonymous or induces a premature termination of the protein), the effect on the translated protein may be significant. In genes intolerant to loss-of-function variants, protein-truncating variants are often associated with diseases and functional consequences ^44^. If a disease is associated with a single penetrant variant or with several variants in the same gene, less effort is required to find the association. However, diseases can be due to a combination of alleles with incomplete penetrance, often across several genes ^45^; a clear association may be difficult to determine in this case. This situation is especially difficult when the variants are observed at a low frequency, as the power to detect an association is higher when variants are common ^45, 46^.

Standard statistical genetic techniques, such as the chi-squared test, Fisher’s exact test, or logistic regression, are often not able to detect associations of a very low frequency variant with a disease phenotype and this is simply because of lack of observations in a sample cohort. However, by aggregating these variants within a given genomic window or functional region (for example, a gene), variants that might individually cause or generate risk for a disease are used to generate a “gene-based association” signal. Such statistical tests, such as the sequence kernel association test (SKAT) ^47^, combined multivariate and collapsing (CMC) ^48^, or custom enrichment algorithms ^49^, combine the signal from individual variants of a given interval into a single score that can be tested against a null model (generally the same score in unaffected controls).

In a study of underlying genetic risk factors for generalized epilepsy, May *et al*. used multiple collapsing statistical methods to test variants within gene sets, namely CMC and SKAT (optimal) ^50^. Although epilepsies tend to have high heritability ^51^, few genetic associations have been found. As with other complex disorders, it may be that several concurrent variants are required to increase risk or that variants are rare and perhaps unique to each phenotypic subset. Even if the study by May *et al*. did not find a single gene that had an enrichment of variants in epilepsy cases, the authors observed a significant association of variants in genes that encode for GABAA receptors ^37^. Only by grouping several genes together into a “gene set” were the authors able to find an association signal; this suggests that any or all of the genes within the set might be associated with the phenotype but that individual variants within genes are too rare or not sufficiently penetrant to generate associations ^50^.

Although this example highlights the success of collapsing statistics to study rare variants, caution must be stressed in terms of limitations. In order to reach the required power both to observe enough rare variation and to detect a substantial difference between cases and controls, considerable sample sizes are required ^44^. Furthermore, these sample sizes depend on phenotypic severity and the relative risk to be detected ^52^. In addition, such findings are of limited value for individual diagnosis and predictive testing.

## Shortcomings of exome sequencing and targeted amplified sequencing

The major downside to ES is the unanalyzed portion of the genome. Increasingly, regulatory, intronic, and intergenic regions are being considered for relevance in biology and disease ^53– 56^. Although there exist some ES kits that enrich for regions outside the strict definition of the exome, a considerable amount of genomic variation is simply not targeted by standard ES. Typically, ES does not target regions of the genome outside of coding exons, although variants within 200 base pairs (bp) of the coding region can be informative variants ^53^.

Disease genetics research and genetic diagnosis will likely move away from ES once cost and ease of analysis for GS are acceptable. A similar number of variants in coding regions are captured by both ES and GS, and generally the variants observed are of equal or higher sequencing quality ^57^. Included in GS results are non-coding variants, those outside of gene-coding regions. However, despite significant effort to categorize the effect of non-coding variants, the effect of non-coding variants generally is not as well known as that of protein-altering exonic variants ^4^. Nonetheless, GS and other extra-exonic technologies are becoming more widely used in genetic disease research.

## Variant burden

Burden studies using ES have focused mainly on the amount of potentially deleterious variants across samples. However, much of the variation in the genome is not apparently deleterious, and although generally there is not a singular cause of disease, these variants might be enriched under certain phenotypes. Although non-coding variants generally do not alter protein structure or function, they may be critical to splicing, regulation, isoform usage, or other functions ^53, 54, 56, 58^. Variants altering the coding sequence of an RNA/DNA-binding protein may affect the affinity of the protein to bind its targets ^59^. Conversely, if the target of one of these binding proteins is altered, a similar loss of affinity might occur ^60^.

An example of the efficacy of GS to study non-coding variation outside the exome is shown in CTCF/cohesin-binding site variants ^55^. Somatic variants occur throughout the genome as a function of DNA replication and repair ^61^; however, the signature and location of these variants may be informative to disease genetic etiology. No single variant in these binding sites was causal per se for colorectal cancers. However, when the focus was on variants across the genome that fall within regions where CTCF and cohesin bind to DNA, a striking number of somatic variants were observed at specific points of the binding sites. This finding was further used to differentiate between subsets of these tumors: the aggregation of somatic variants within CTCF-binding sites was observed only in microsatellite-stable tumors, whereas microsatellite-instable and *POLE* variant-related tumors showed no such enrichment. This finding could be generalized to any transcription factor– or DNA–binding sites ^60^ but would require GS, as ES would not sequence many of the binding sites.

## Copy number variation detection

Many copy number variation (CNV) callers have been created for ES data to varying degrees of accuracy and usage ^62^. A recent refinement of ES CNV calling showed that information from multiple callers and quality-control metrics could increase ES CNV accuracy substantially ^63^. However, CNV detection will suffer from the limited target regions of ES ^64^. Furthermore, more sophisticated tools accurately resolve small CNV across the genome, using deviations from GS coverage uniformity ^65^ or using reads that align to a given motif sequence ^64^.

The shortcomings of ES to find repetitive pathogenic variants can be seen in the identification of repeat expansions. A striking example is the recent discovery of a novel genetic cause of glutamine deficiency ^66^. Three unrelated patients were screened using traditional ES in order to find protein-altering variants in the glutaminase ( *GLS*) gene. As any identified variants were also observed in the unaffected parents, GS was used to test for additional variation near the gene. A trinucleotide repeat expansion in the 5′ untranslated region of *GLS* was identified as a disease-causing variant; patients either carried a non-synonymous *GLS* variant in trans with the expansion or carried two expanded alleles. The expanded repeat resulted in decreased expression of *GLS*, possibly through modified chromatin ^66^. This finding relied on another recent advancement in repeat detection, the software Expansion Hunter ^64^, which allows local discovery and estimation of repeat sequence deviation using observed repeats within sequencing reads.

This example highlights the added benefit of studying the genome outside the exome. A variant of incomplete segregation or penetrance was observed, and had the study been conducted prior to the ability to perform GS, this might have been the conclusion of the study. However, by analyzing outside the regions targeted in ES, a non-coding repeat variant was able to explain the disease in these patients and explain the inability of the missense variant to cause disease alone ^66^.

## Long-read sequencing

There are genomic regions that owing to technical limitations are not studied and that could have implications for clinical genetics ^67^. These regions could be categorized as difficult to sequence because of nucleotide composition and as possible to sequence but difficult to confidently place in the genome because of sequence complexity ^67^. Both ES and GS rely on the speed and ease of generating fragmented genomic sequences that can be aligned back to the reference genome. However, these genomic regions that are difficult to sequence or align create major problems in fully assessing genetic risk, as they are not included in association studies or clinical genomics ^68^. Furthermore, genotyping errors can result in ES if large structural variants (SVs) are not detected by short-read sequencing ^69^. SVs themselves are important with regard to disease etiology: ES and GS have difficulty resolving both simple SV and complex SV (multiple genomic breakpoints) and these variants can have marked effects on individual disease progression ^70^.

The genetic etiology of an unknown number of diseases might lie in these genomic regions that are difficult to examine. GS and ES can estimate the probable number of repeats in a repeat expansion but this is by inference of the number of reads with a repetitive sequence and the composition of the region of interest ^64^. Because the repeat expansion can exceed the number of bases in a short read (typically between 50 and 150 bp), the repeat is not directly observed. LRS allows direct study of high-molecular-weight DNA samples ^71^ either through recording current changes induced by the passage of a DNA molecule through a channel (Oxford Nanopore Technologies, Oxford, UK) ^72^ or through imaging of an anchored polymerase and fluorescent nucleotide additions (PacBio, Menlo Park, CA, USA) ^73^. Both technologies allow the sequencing of several kilobase-long genomic fragments ^72, 73^.

A more recent example of the utility of LRS of an intronic repeat expansion is that of the gene sterile alpha motif domain-containing protein 12 ( *SAMD12*) in familial cortical myoclonic tremor with epilepsy (FCMTE) ^74, 75^. Several loci associated with FCMTE have been reported which were found through classic linkage studies and ES ^76^. Despite the existence of several genes in each locus, no causal variants had been discovered for one of the loci (FCMTE1), even after having applied ES ^76^. Using nanopore sequencing, two independent groups were able to describe an expanded repeat and insertion of a separate motif within the *SAMD12* gene that associated with FCMTE1 ^74, 75^. This repeat expansion would not be found using ES, as it was intronic and not covered by conventional ES. CNV detection methods in GS may not have observed the expansion, as repetitive reads did not align well to the reference and were problematic to test using polymerase chain reaction (PCR) ^74^.

Although LRS has helped our understanding of regions that are difficult to test using conventional ES or GS, it has been used mainly in a targeted manner ^77– 79^. The throughput of this technology does not allow deep, high-fidelity sequencing of the entire human genome, and repeat discovery methods using long-read genome sequencing are lacking. However, as technologies become more refined in the future, high-depth and high-fidelity LRS may become the new standard for ES or GS analyses.

## Conclusions

A plethora of ES data has been generated in the past decade. New technologies and sequencing methods will eventually supplant ES, but the data it generated will continue to be useful in disease research. With the shift to more collaborative and open science, smaller-scale ES studies will contribute to large-scale consortium ES studies, using very large cohort sizes to detect very rare variants. The success of this paradigm shift has been seen in undertakings such as the Simons Simplex Collection ^80^, the ALS exome collaboration ^81^, or the UK Biobank ^82^.

Despite its shortcomings, ES will continue to be used in disease research and its applications. Its ease of generation and interpretation allow rapid analysis. Until GS can be performed and analyzed for equal or lesser cost than ES and unless significant progress is made in understanding non-exonic variants, ES will continue to be used to study disease. Clinical screening of potential genetic causes of disease can be easily performed using ES. As our knowledge of the exome remains incomplete, we will continue to study the protein-coding regions. However, much variation outside of the exome must account for genetic causes of disease and therefore research must strive to understand the entire genome.

## Abbreviations

ALS, amyotrophic lateral sclerosis; CMC, combined multivariate and collapsing; CNV, copy number variant; ES, exome sequencing; FCMTE, familial cortical myoclonic tremor with epilepsy; GLS, glutaminase; GS, genome sequencing; LRS, long-read sequencing; SAMD12, sterile alpha motif domain-containing protein 12; SKAT, sequence kernel association test; SV, structural variant
